# In situ breast cancer incidence patterns in Iceland and differences in ductal carcinoma in situ treatment compared to Sweden

**DOI:** 10.1038/s41598-020-74134-5

**Published:** 2020-10-19

**Authors:** Arnar S. Agustsson, Helgi Birgisson, Bjarni A. Agnarsson, Thorvaldur Jonsson, Hrefna Stefansdottir, Fredrik Wärnberg, Mats Lambe, Laufey Tryggvadottir, Asgerdur Sverrisdottir

**Affiliations:** 1 Faculty of Medicine, University of Iceland, Reykjavík, Iceland; 2 Landspitali, The National University Hospital of Iceland, Reykjavík, Iceland; 3 Icelandic Cancer Registry, Icelandic Cancer Society, Reykjavík, Iceland; 4 Regional Cancer Centre Uppsala-Örebro, Uppsala, Sweden; 5 Department of Medical Epidemiology and Biostatistics, Karolinska Institutet, Stockholm, Sweden; 6 Sahlgrenska Academy, Gothenburg University, Göteborg, Sweden

**Keywords:** Cancer, Epidemiology, Population screening

## Abstract

The purpose was to review the incidence of in situ carcinoma in Iceland after initiating population-based mammography screening in 1987 and to compare management of ductal carcinoma in situ (DCIS) between Iceland and the Uppsala–Örebro region (UÖR) in Central Sweden. The Icelandic Cancer Registry provided data on in situ breast carcinomas for women between 1957 and 2017. Clinical data for women with DCIS between 2008 and 2014 was extracted from hospital records and compared to women diagnosed in UÖR. In Iceland, in situ carcinoma incidence increased from 7 to 30 per 100 000 women per year, following the introduction of organised mammography screening. The proportion of in situ carcinoma of all breast carcinomas increased from 4 to 12%. More than one third (35%) of women diagnosed with DCIS in Iceland were older than 70 years versus 18% in UÖR. In Iceland, 49% of all DCIS women underwent mastectomy compared to 40% in UÖR. The incidence of in situ carcinoma in Iceland increased four-fold after the uptake of population-based mammography screening causing considerable risk of overtreatment. Differences in treatment of DCIS were seen between Iceland and UÖR, revealing the importance of quality registration for monitoring patterns of management.

## Introduction

Mammography screening programs contribute to the increasing incidence of in situ carcinomas in the breast^1, 2^. The majority of these lesions consists of ductal carcinoma in situ (DCIS) which is a localized cancerous formation of the epithelial cells in the mammary duct^2^. Women diagnosed with in situ breast cancer are at increased risk of a subsequent diagnosis of invasive breast cancer and the risk is considerably higher for those with positive family history and in younger women^3^. In Iceland, nationwide population-based mammography screening was introduced in 1987 for women aged 40–69 years, at a two-year interval.

DCIS is treated surgically, either with mastectomy or breast conserving surgery (BCS). BCS is generally considered the first choice in early stage breast cancer and in situ carcinomas, and is not associated with reduced long-term survival compared to mastectomies^4,5^. European guidelines from the ESMO group recommend that 60–80% of primary breast cancer patients should undergo BCS^6^.

When undergoing BCS instead of a mastectomy for DCIS, there is an increased risk of local relapse and therefore adjuvant radiation therapy may be indicated. Radiation therapy after BCS reduces the risk of local recurrence by 46% over ten years follow-up compared to BCS alone, resulting in similar recurrence rates as after a mastectomy^7,8^. Radiation therapy is indicated after BCS, but might be omitted if the risk of recurrence is low, such as in low grade DCIS, small tumour size and with adequate surgical margins.

Sentinel lymph node biopsy (SLNB) is performed in DCIS surgery since about 20% of women with DCIS are found to have invasive carcinoma in the surgical specimen. The risk for positive SLNB in DCIS tumours only is low^9^.

The Swedish National Quality Registry for Breast Cancer (SQR) was made available on a nationally uniform platform in 2008, allowing for the comparison of breast cancer management between regions. The SQR reports also include comparisons between hospitals within the different regions, as the Uppsala–Örebro region (UÖR) in Central Sweden. In Iceland, quality registration has recently started, using forms that are based on the Swedish model.

The purpose of this study was to describe the incidence of in situ carcinoma in Iceland and to compare the management of DCIS between Iceland and UÖR in Sweden.

## Materials and methods

The Icelandic Cancer Registry provided data on the incidence of in situ breast cancer in women during the time period 1955–2017^10^. Age standardised incidence rates, using the World standard, including 95% confidence intervals, were calculated for the mammography screening target group 40–69 years. The proportion of in situ carcinomas of all carcinomas was calculated for the age group 40–69 years and the age groups 40–49, 50–59 and 60–69 years old.

For the comparison with UÖR, the Icelandic Cancer Registry provided a list of all women (n = 136) diagnosed with in situ carcinomas in Iceland from the year 2008 through 2014. Women with a prior diagnosis of invasive breast cancer (n = 19) or lobular carcinoma in situ (n = 7) were excluded, resulting in 110 women diagnosed with a primary DCIS.

A further analysis of the Icelandic cohort was performed in order to find all women upstaged during the study period from DCIS to invasive cancer. All women with invasive breast cancer diagnosed 2008–2014 were examined in Iceland, finding all women who were presumed to have DCIS at the time of initial surgery but were later upstaged to invasive cancer. A total of 16 women had been upstaged in the time period, due to findings of an occult invasive component in the surgery sample or a positive lymph node. Those 16 women were analyzed separately and not included in the overview of the Icelandic cohort nor in the comparison to UÖR.

At Landspítali, the National University Hospital of Iceland, registration forms had been constructed in the data management system (Heilsugátt), which is a part of the electronic hospital patient record system. The registration forms containing variables of diagnosis, treatment and follow-up of patients with breast cancer were translated and adapted from the registration forms of the SQR^11^. This register is built on the platform of *Informationsnätverk för cancervården* (INCA), used for all Swedish National Quality Registers. INCA has been in use for breast cancer since 2008.

After the registration forms had been completed, the dataset was encrypted and personal identifiers removed. Selected variables were sent to Sweden for comparisons with prospectively registered patients at the Regional Cancer Centre in UÖR during the same time period. Stata/IC 14.1 was used to calculate the Chi-squared test for comparing proportions. All statistical tests were two-sided and P-values < 0.05 were considered to be statistically significant.

The study was approved by the National Bioethics Committee of Iceland (VSN-17-003). According to Icelandic law, article 11 in 90/2018 regarding Act on Data Protection and the Processing of Personal Data, and with the approval of the National Bioethics Committee of Iceland, further patient consent is not required as the data is depersonalized and many of the patients are deceased.

### Ethical standard

This article does not contain any studies with human participants or animals performed by any of the authors.

## Results

**Figure 1:**
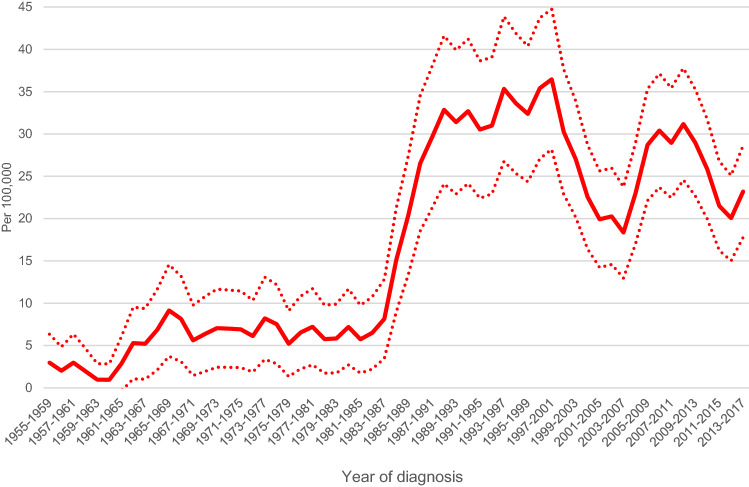
Age standardized (World) incidence of in-situ breast cancer in Iceland in 40–69 years old women during the time period 1955–2017 including 95% confidence interval, five-year moving averages.

Total of 438 women aged 40–69 years old were diagnosed with DCIS in Iceland from 1955 to 2017. The yearly average number was 1.5 during 1955–1987, but thereafter it was 13. The age-standardized incidence rate (five-year moving averages) for in situ breast carcinomas is presented in Fig. 1. A sharp rise in the incidence of in situ breast carcinoma was seen after the onset of population-based mammography screening in 1987, from around 7 per 100 000 to 30 per 100 000 inhabitants per year. The confidence intervals before and after onset of the screening programme do not overlap, indicating a statistically significant difference in rates.

**Figure 2:**
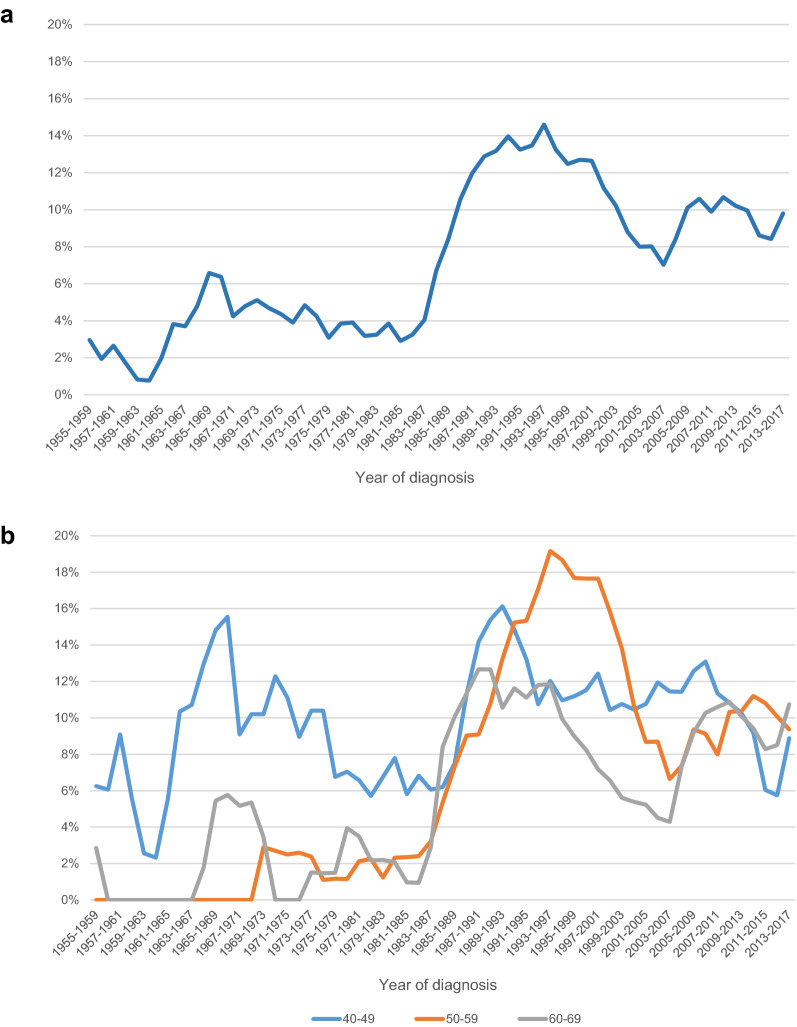
Proportion of in-situ breast cancer to in-situ and invasive breast cancer in 40–69 years old women during the time period 1955–2017 (**a**) and comparison of the age groups 40–49, 50–59 and 60–69 years (**b**).

Similarly, a marked increase was seen after 1987 in the proportion of in situ carcinomas of all breast carcinomas diagnosed at ages 40–69 years from 4 to 12% (Fig. 2a) with a peak of 19% in the 1990s for those 50–59 years old (Fig. 2b).

Before 1987, the highest proportion of in situ carcinomas of all breast cancer was observed in the youngest age group (40–49 years). In this age group, there was no apparent increase in the proportion after 1987, with the proportion circulating around 10% for the entire period 1957–2017 (Fig. 2b). The sharpest rise in the proportion of in situ carcinomas of all breast carcinomas after 1987 was seen for ages 50–59 years, from 2 to 18%, although lowering to 10% in the latest decade (Fig. 2b). For ages 60–69, the increase was from 2 to 10% (Fig. 2b). Random variation is prominent in all age groups due to the low number of cases.

A total of 110 women were diagnosed with a primary DCIS in the period 2008–2014 in Iceland, representing 8.1% of all breast cancer diagnoses in women. Patient characteristics and an overview of DCIS diagnosis in Iceland are shown in Table 1. Of the women who underwent mastectomy, 46% had direct reconstruction of the breast. SLNB was done in 95% of women who had mastectomies and in 42% of women with BCS. Seventy-three percent of the tumours among women undergoing mastectomy had nuclear grade three, compared to 50% in BCS.

**Table 1:** Patient characteristics and DCIS diagnosis in Iceland 2008–2014.

	Proportion (%)
No of women	110
Median age (range)	64 years (31–100)
Diagnostic imaging	105 (96%)
Tissue biopsy (core needle or surgical)	110 (100%)
Fine needle aspiration	90 (82%)
Clinical signs of tumour	25 (23%)
DCIS diagnosis confirmed before first hospital interview	85 (77%)
Screening detected	90 (82%)
DCIS diagnosis confirmed before surgery	90 (82%)

Comparison of clinical, pathological and treatment related factors, in women diagnosed with DCIS in the years 2008–2014, between Iceland and UÖR is presented in Table 2. In Iceland, 35% of women were 70 years or older versus 18% in UÖR (*p* =  < 0.001). Differences in nuclear grade and in the use of pre- and postoperative multidisciplinary meetings were also seen. In Iceland direct reconstruction after mastectomy was performed in 50% of women compared to 19% of women in UÖR.

**Table 2:** Comparison of clinical-, pathological and treatment related factors, in women diagnosed with ductal carcinoma in situ 2008–2014 in Iceland and Uppsala Örebro Region (UÖR).

	Iceland	UÖR	*p*-values*
No of patients	110	1130	
< 60 years old	36 (33%)	520 (46%)	< 0.001
60—69 years old	35 (32%)	397 (35%)	
70—79 years old	31 (28%)	163 (14%)	
80 + years old	8 (7%)	50 (4%)	
Patients 45–69 years old diagnosed through screening	78/90 (87%)	822/1023 (80%)	
Nuclear grade 1	18 (17%)	96 (9%)	0.004
Nuclear grade 2	25 (23%)	366 (36%)	
Nuclear grade 3	64 (60%)	493 (48%)	
Nuclear grade not evaluated	0	62 (6%)	
Tumor size over 15 mm	57/93 (61%)	672/1055 (64%)	
Confirmed pre-operative DCIS diagnosis	90/110 (82%)	825/1130 (73%)	
Sentinel lymph node biopsy	63 (57%)	603 (54%)	
Lymph node dissection	4 (4%)	28 (2%)	
Axillary surgery not performed	41 (37%)	482 (43%)	
Pre-operative multidisciplinary meeting	48/101 (48%)	1051/1124 (94%)	< 0.001
Post-operative multidisciplinary meeting	99/110 (90%)	1096/1122 (98%)	< 0.001
Mastectomy	54 (49%)	446 (40%)	
Breast conserving surgery (BCS)	56 (51%)	675 (60%)	
Radiotherapy after BCS (% of BCS)	30/56 (54%)	438/675 (65%)	

*Only significant *p*-values shown.

No positive lymph nodes were found in the 63 SLNB in Iceland. Of the 16 women upstaged from DCIS, seven underwent a SLNB alongside initial surgery while nine did not. Of the seven, two had positive lymph nodes. Of the total number of cases initially presumed of DCIS and who underwent SLNB, 97% (68/70) did not have a positive lymph node during the study period.

Eight patients with positive lymph nodes were registered in UÖR, five were from axillary dissection resulting in three women with positive lymph nodes among the 603 women in UÖR who underwent SLNB.

Adjuvant endocrine therapy was prescribed for 2% of women in UÖR and 4% in Iceland.

## Discussion

The present study spanning a 60-year period, demonstrated a four-fold, sharp increase in the incidence of in situ breast cancer after the introduction of population-based mammography screening in 1987 in Iceland. This increase suggests that the screening has introduced some overdiagnosis of in situ breast cancer.

The results from the comparison on diagnosis and treatment of DCIS between Iceland and UÖR demonstrated a statistically significant difference in age of the women diagnosed, in nuclear grade and in the frequency of multidisciplinary meetings. Interesting, although not statistically significant, was the higher use of mastectomies and less frequent application of adjuvant radiation therapy after BCS in Iceland compared with UÖR.

Studies from the USA, France and Norway have also demonstrated an increase in the incidence of in situ breast cancer after the initiation of mammography screening programs, especially in women aged 50 years or older^12–14^. Another interesting finding is that the increase in women over 50 years old seems to be mainly in less-aggressive histological types of DCIS^1^.

It is assumed that the majority of in situ breast cancer lesion will not develop into invasive breast cancer. This was supported by a study of 45 women with low-grade DCIS treated with biopsy only and observed over 47 years, showed that over time, 36% developed invasive breast cancer^15^. Therefore, there is a risk of substantial overtreatment of women diagnosed with low-grade DCIS, which may affect their quality of life. Tools for better individual risk assessment that would minimize unnecessary treatments are thus needed^16^.

The rise in incidence of in situ carcinomas of the breast and subsequent lack of reduction in invasive breast cancer incidence is among the reasons for the current debate concerning the benefit to harm ratio in population screening programs. Gabe et al.^17^, analysed the efficacy of the Icelandic national screening program, estimating around 40% mortality reduction from breast cancer among women attending the screening.

The amount of overdiagnosis associated with screening for breast cancer has been highly debated and results differ substantially between studies. Jorgensen et al., estimated the effects of population breast cancer screening in Denmark and concluded that one out of three diagnoses of breast cancer were overdiagnosis^18^. However, another Danish study estimated that for every two to three prevented breast cancer deaths merely one was overdiagnosed^19^. In Denmark, studies have estimated that only around 1% of screened breast cancer diagnoses were overdiagnosis and that the breast cancer mortality was reduced by 25% in the screening period and 37% for women attending screening^20^. Similarly, in the EUROSCREEN trial, four cases of overdiagnosis were made per 1000 women compared to seven to nine lives saved^21^.

In Iceland, the target age groups for the population-based mammography screening have been 40–69 years from the outset. However, there is limited evidence for benefits of mammography screening at ages under 50 years, except for selected genetic subgroups^22^. The incidence of in situ carcinomas quadrupled in 1987 at the beginning of population breast cancer screening. It is of interest that the increased proportion of in situ carcinoma to all breast cancer was seen mainly for women over 50 years of age and not for women under age 50. However, the numbers are very small and random variation prominent. In situ carcinomas represent around 8% of all breast carcinomas today in Iceland.

The proportion of women age 70 years or older at DCIS diagnosis was higher in Iceland than in Sweden. This was unexpected as the mammography screening program in Sweden includes 70–74 years old women. However, in Iceland, symptom-free women over 69 years who wish to attend mammography screening, are accepted for mammography even though they are not formally invited. We do not have the numbers on how common that is in Iceland.

A difference in DCIS nuclear grade was also observed between the regions. Compared to UÖR, Iceland had statistically significantly higher proportion of women with DCIS grade one and with grade three and a lower proportion with grade two. At least a part of these differences may be explained by differences in grading practices between Iceland and UÖR. Some of the Icelandic pathologists namely grade DCIS as being of either low grade (grade one) or high grade (grade three) with grade two DCIS thus being underrepresented in the Icelandic data (Agnarsson BA, personal communication). Sweden uses the universally recognized NG system of classifying DCIS in grade one to three^23,24^. The authors conclude that this difference in grading is likely due to the approach to grading between the regions and unlikely represents a true difference in pathology of the cancers.

Multidisciplinary meetings were less common in Iceland than in UÖR and the difference was most striking concerning pre-operative meetings. A breast cancer center has been established at Landspitali University Hospital during the time period of this study and multidisciplinary meetings have become a routine. After the establishment of a breast cancer center in Iceland, pre-operative multidisciplinary meetings did increase. Recent study on the quality registration of invasive breast cancer in Iceland 2016–2017 revealed a significant increase in the use of multidisciplinary meetings with 98% of the cases discussed preoperatively and 99% postoperatively at the multidisciplinary meetings^25^.

The reasons for the relatively high proportion of mastectomies in Iceland and the increase in mastectomies in UÖR are not clear. Interestingly, the proportion of DCIS patients with mastectomy has been increasing in Sweden during the past 20 years^26^. The high prevalence of mastectomies in Iceland cannot be explained by our data or previous Icelandic studies. However, the easy access to immediate breast reconstruction, increased usage of MRI in diagnosis, increasingly available results from genetic testing and the patient’s own choice might partly be causing a high prevalence in Iceland since these factors are causing an increase in mastectomies in other Western countries.

 As pre-operative MRI is a standard at the University Hospital in Iceland^27^, that might be an important reason for the high proportion of mastectomies in Icelandic DCIS patients. According to results from a recent meta-analysis, women who had pre-operative MRI were 70% more likely to undergo a mastectomy than BCS as initial surgery^28^. However, data on pre-operative MRI was not available in this study and therefore this only remains a hypothesis.

In Iceland, the non-significant higher proportion of mastectomies might reflect differences in preference of type of surgery, as a similar group of women undergoing BCS and radiotherapy in UÖR would have undergone mastectomies in Iceland. Immediate reconstruction of the breast has become increasingly available in the last few years as oncoplastic techniques have improved substantially. Recent research into the quality of life shows that immediate breast reconstruction after a mastectomy gives similar results as BCS in terms of quality of life^29^.

The low rate of radiotherapy observed after BCS in Iceland was unexpected. The guidelines recommend radiation therapy after BCS in most cases of DCIS. The proportion of women with DCIS who are treated with BCS in Iceland is lower than observed in other Western countries where close to two-thirds of patients undergo BCS^30–33^. Surgical guidelines from the Association of Breast Surgery at BASO state that mastectomies should only be performed under certain indications, such as large tumours and extensive microcalcifications^34^. This is further supported by the ESMO guidelines, stating that BCS with oncoplastic techniques is the mainstay of surgical treatment in primary breast cancer^6^.

Surgical preference towards mastectomies in Iceland would result in an increase in mastectomies and fewer BCS. Subsequently, proportionately fewer women should have undergone radiotherapy as larger and high-grade DCIS would have been selected for mastectomies in Iceland. While the group with smaller and low-grade DCIS would have still undergone BCS. Therefore, radiation therapy would be needed less often. That might explain the proportionally lower rates of radiation therapy in Iceland.

SLNB was performed in a large proportion of the women both in UÖR and in Iceland. In both Iceland and UÖR, a full axillary lymph node dissection was performed only in a few surgeries. That type of axillary surgery for DCIS is considered outdated and is not recommended today^6,34–36^. The BASO guidelines even state that lymph node dissection in DCIS is contraindicated^34^.

One difference between the regions is that in Iceland, the Icelandic Cancer Registry would have upstaged any DCIS with a positive sentinel lymph node to invasive breast cancer. This was not done for the few cases of DCIS with positive sentinel lymph node in UÖR. A consensus between cancer registries would be preferable on how to register these patients.

Current SLNB might be on the way out. A recently published Swedish study showed a new promising approach to SLNB by using superparamagnetic iron oxide, enabling SLNB to be performed later only on those with invasive cancer after tumour resection and allowing up to 80% of women to avoid SLNB altogether^37^. Four ongoing trials, LORD, LORETTA, COMET and LORTIS are independently assessing active surveillance and/or endocrine therapy as an alternative non-inferiority treatment to low-risk DCIS^38–41^. The implications are that an equally safe but less harmful treatment alternative might be on the horizon, especially for patient groups with low risk DCIS and of older age.

Considering the sharp rise in incidence especially for women over 50 years old, high proportion of mastectomies and SLNB and the lower risk of invasive recurrence in older women, the group of women ages 50 or older appears to be at a significant risk of overtreatment with a DCIS diagnosis.

The strength of this study was that systematic comparisons between countries were made possible by use of almost identical population-based quality registration forms using the same variable definitions. Additional strengths of our study included the use of population-based data from high quality cancer registries with information retrieved from all breast cancer units in both regions. A long-standing cancer register in Iceland allowed for the assessment of over 60 years of in situ incidence. The quality registration for diagnoses and treatment was prospective in UÖR and retrospective in Iceland.

Weaknesses included the small size of the Icelandic population, resulting in large random fluctuations in rates, which are especially prominent for rare cancers such as DCIS with 16 women diagnosed annually on the average between 2008 and 2014. In retrospect, the registry forms used in Iceland were not fully compatible at the time and therefore a few variables were left out. The registration form has been updated and is at present time fully compatible to the Swedish registration.

In conclusion, the incidence of in situ carcinoma increased four-fold in Iceland following the introduction of population-based mammography screening. The increase was more pronounced in the group of women aged 50 years or older, this group might therefore be at higher risk of overtreatment than younger women attending mammography screening.

Differences in treatment of DCIS were seen between Iceland and UÖR, revealing the importance of systematic registration of clinical management that allows for monitoring of adherence to guidelines and facilitates identification of areas in need of improvement.

## Data Availability

The data that support the findings of this study are available from the Icelandic Cancer Registry for the Icelandic data and the Regional Cancer Centre Uppsala–Örebro for the UÖR data but restrictions apply to the availability of these data, which were used under license for the current study, and so are not publicly available. Data are however available from the authors upon reasonable request and with permission of ethical committees in Iceland and Sweden and the Regional Cancer Centre Uppsala–Örebro and the Icelandic Cancer Registry.
